# Severity of Liver Fibrosis Is Associated with the Japanese Diet Pattern and Skeletal Muscle Mass in Patients with Nonalcoholic Fatty Liver Disease

**DOI:** 10.3390/nu15051175

**Published:** 2023-02-26

**Authors:** Yoshinari Matsumoto, Hideki Fujii, Mika Harima, Haruna Okamura, Yoshimi Yukawa-Muto, Naoshi Odagiri, Hiroyuki Motoyama, Kohei Kotani, Ritsuzo Kozuka, Etsushi Kawamura, Atsushi Hagihara, Sawako Uchida-Kobayashi, Masaru Enomoto, Yoko Yasui, Daiki Habu, Norifumi Kawada

**Affiliations:** 1 Department of Nutrition, Graduate School of Human Life and Ecology, Osaka Metropolitan University, 3-7-30 Habikino, Habikino-shi, Osaka 583-8555, Japan; 2 Department of Hepatology, Osaka Metropolitan University Graduate School of Medicine, 1-4-3 Asahimachi, Abeno-ku, Osaka 545-8585, Japan; 3 Nutrition Department, Osaka Metropolitan University Hospital, 1-5-7 Asahimachi, Abeno-ku, Osaka 545-8586, Japan; 4 Department of Premier Preventive Medicine, Osaka Metropolitan University Graduate School of Medicine, 1-4-3 Asahimachi, Abenoku, Osaka 545-8585, Japan

**Keywords:** Japanese diet, nutrition, lifestyle, liver fibrosis, nonalcoholic steatohepatitis, muscle mass

## Abstract

It is not fully clear as to which dietary patterns are associated with the pathogenesis of nonalcoholic fatty liver disease (NAFLD) in Asia. We conducted a cross-sectional study of 136 consecutively recruited patients with NAFLD (49% female, median age 60 years). Severity of liver fibrosis was assessed using the Agile 3+ score, a recently proposed system based on vibration-controlled transient elastography. Dietary status was assessed using the 12-component modified Japanese diet pattern index (mJDI12). Skeletal muscle mass was assessed by bioelectrical impedance. Factors associated with intermediate–high-risk Agile 3+ scores and skeletal muscle mass (75th percentile or higher) were analyzed by multivariable logistic regression. After adjustment for confounders, such as age and sex, the mJDI12 (OR: 0.77; 95% CI: 0.61, 0.99) and skeletal muscle mass (75th percentile or higher) (OR: 0.23; 95% CI: 0.07, 0.77) were significantly associated with intermediate–high-risk Agile 3+ scores. Soybeans and soybean foods were significantly associated with skeletal muscle mass (75th percentile or higher) (OR: 1.02; 95% CI: 1.00, 1.04). In conclusion, the Japanese diet pattern was associated with the severity of liver fibrosis in Japanese patients with NAFLD. Skeletal muscle mass was also associated with the severity of liver fibrosis, and intake of soybeans and soybean foods.

## 1. Introduction

Nonalcoholic fatty liver disease (NAFLD) is a type of liver disease that includes simple fatty liver and steatohepatitis, not caused by excessive alcohol consumption [1]. The pathogenesis of nonalcoholic steatohepatitis (NASH) with inflammation of the liver tissue is important in NAFLD, because progression of NASH leads to cirrhosis, liver failure, and the risk of hepatocellular carcinoma [2,3,4,5,6]. NAFLD is also associated with an increased risk of cardiovascular-disease-related mortality [7,8] and total mortality [2,5]. Some expert reviews have shown that body weight loss is the only remedy that prevents the progression of NAFLD [9,10] and that body weight loss by lifestyle modification, including diet and exercise, is desirable [11,12]. Other factors, such as diet quality and composition, have been associated with the progression of NAFLD [13]. The Mediterranean diet pattern—which consists mainly of plant foods and olive oil, and relatively few animal products—has been shown to reduce hepatic fat accumulation and insulin resistance in an intervention study of patients with NAFLD [14], and observational studies have also reported that an increase in Mediterranean diet score—which indicates a dietary pattern similar to the Mediterranean diet—is associated with an improvement in measures of liver fat mass in patients with NAFLD [13]. However, from the perspective of food culture, it may be difficult to promote the Mediterranean diet pattern to the Asian region, including Japan. It is also not fully clear as to which dietary patterns are associated with the pathogenesis of NAFLD in Asia. The Japanese diet pattern (JD) is a diet pattern unique to Japan, that consists of soybeans and soybean foods, fish and shellfish, vegetables, mushrooms, seaweed, green tea, and fermented foods, such as pickles and miso [15]. In Japan, dietary intervention with the Japanese diet pattern showed positive effects on weight loss and associated improvements in serum lipid metabolism, and it is expected to improve health maintenance [16]. However, to our knowledge, there are no reports on the relationship between the Japanese diet pattern and NAFLD.

Muscle is an important tissue involved in glucose metabolism, and a decreased skeletal muscle mass is associated with impaired glucose tolerance [17]. As impaired glucose tolerance is a risk factor of NAFLD [18], maintenance of skeletal muscle mass may be important in the management of the condition in patients with NAFLD. In addition, sarcopenia—a loss of skeletal muscle mass and muscle function—is a risk factor for NAFLD independent of insulin resistance [19], suggesting a possible muscle–liver organ interaction even outside of glucose metabolism. However, the association between the severity of liver fibrosis in NAFLD and skeletal muscle mass has not been fully validated. In addition, dietary factors affect skeletal muscle mass, but the relationship between skeletal muscle mass and dietary factors in patients with NAFLD is unclear.

Therefore, this study aimed to examine the relationships between the Japanese diet pattern and the severity of liver fibrosis in patients with NAFLD. We also investigated the relationships between skeletal muscle mass, dietary factor, and the severity of liver fibrosis in patients with NAFLD.

## 2. Materials and Methods

### 2.1. Study Subjects

**Figure 1:**
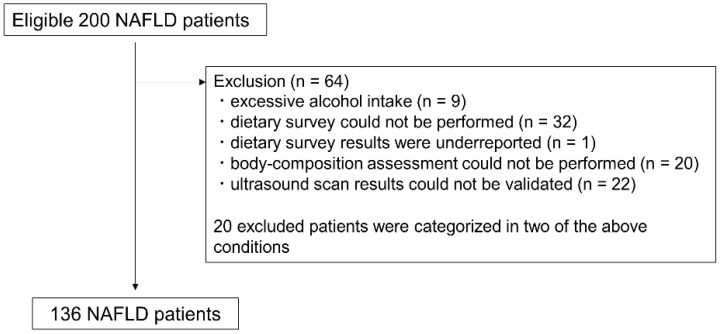
Study subject flowchart. NAFLD: Nonalcoholic fatty liver disease.

We recruited 200 patients with suspected fatty liver on ultrasonography from September 2021 to November 2022 for a prospective cohort study of patients aged 20 years or older who attended the Department of Hepatology, Osaka Metropolitan University Graduate School of Medicine. We performed a cross-sectional analysis using baseline data. Of the 200 patients, we excluded 9 patients with excessive alcohol intake (>30 g/day in male; >20 g/day in female) [20], 32 patients for whom a dietary survey could not be performed, 1 patient whose dietary survey results were underreported, 20 patients for whom body composition assessment could not be performed, and 22 patients whose ultrasound scan results could not be validated. Because 20 excluded patients were categorized in two of the above conditions, 64 patients were excluded and 136 patients were included in the final analysis (Figure 1). In accordance with the Declaration of Helsinki, written informed consent to participate in the study was obtained from all patients prior to the start of the study. The study was approved by the Ethics Committee of Osaka City University (now known as Osaka Metropolitan University) School of Medicine (approval number: 2021-088; approval date: 17 June 2021).

### 2.2. Patient Characteristics

Data were extracted regarding body mass index (BMI), daily alcohol intake, past medical history, and current drug history.

### 2.3. Alcohol Intake Screening

Daily alcohol consumption was calculated in grams, using our modified template [21]. Briefly, we assessed drinking frequency (daily, weekly, monthly, or yearly) and the volume of alcohol intake by beverage type (e.g., beer, shochu, Japanese sake, whisky, and wine). The volume of alcohol intake was converted to grams of ethanol, and values for each beverage type were added. The specific density of alcohol was defined as 0.79 g/mL. Alcohol intake greater than 0 g ethanol was defined as “having drinking habits”.

### 2.4. Assessment of Liver Fibrosis Risk

To assess the risk of liver fibrosis, vibration-controlled transient elastography of the liver was carried out with the FibroScan^®^ Mini 430 (Echosens, Paris, France). The FibroScan Mini 430 simultaneously measures the liver stiffness measurement and the controlled attenuation parameter. The controlled attenuation parameter has been designed to measure liver ultrasonic attenuation (go-and-return path) at 3.5 MHz, on both M and XL probes [22]. The final controlled attenuation parameter and liver stiffness measurement results were expressed in dB/m and kPa, respectively. Only examinations with at least 10 valid measurements per patient were accepted for analysis. The risk of liver fibrosis was assessed using the Agile 3+ score, a recently proposed scoring system based on vibration-controlled transient elastography. To calculate the Agile 3+ score, the aspartate aminotransferase/alanine aminotransferase ratio, diabetes status, sex, age, and liver stiffness measurements were used [23]. The Agile 3+ score has been reported to be as predictive of liver-related events in patients with NAFLD as the existing liver fibrosis assessment score, Fibrosis-4 [23,24]. An Agile 3+ score of 0.451 was used as the cut-off value that achieved a sensitivity of greater than or equal to 85% [23], and was also used to divide the patients into two groups: Agile 3+ scores of less than 0.451 (low-risk Agile 3+ score) and 0.451 or higher (intermediate–high-risk Agile 3+ score).

### 2.5. Food and Nutrient Intake Survey and Calculation of mJDI12

Food and nutrient intake status was assessed using a brief-type self-administered diet history questionnaire (BDHQ) [25,26]. The BDHQ is used to assess the approximate intake of food and nutrients for the month prior to the time of the survey. In accordance with previous reports, 1 patient with less than 600 kcal energy intake was excluded as an underestimate [27]. The 12-component modified Japanese diet pattern index (mJDI12) was calculated to evaluate dietary patterns [28]. The mJDI12 is based on the intake of 12 foods or food groups, and consists of the Japanese diet pattern index (JDI) [29] based on the intake of 9 foods or food groups, plus 3 food groups that were considered to contribute to the composition of the Japanese diet pattern by a qualitative systematic review [15,28]. The mJDI12 was calculated based on the intake per 1000 kcal of soybeans and soybean foods, green and yellow vegetables, fruit, fish and shellfish, pickles, mushrooms, seaweeds, green tea, rice, miso soup, beef and pork, and coffee. We scored our patients as either at, above or below the median intake for each food or food group for each sex [28]. For beef and pork and coffee, 1 point was scored for an intake below the median; for all other foods or food groups, 1 point was scored for an intake above the median. The higher the mJDI12 score, the closer the patient’s diet is to a Japanese diet pattern.

### 2.6. Assessment of Body Weight and Body Composition

Measures of body weight and body composition were determined by a vertical direct segmental multi-frequency bioelectrical impedance analysis analyzer (InBody© 270, InBody USA, Cerritos, CA, USA). The InBody© 270 records a user’s weight, skeletal muscle mass, percent of body fat, and BMI, to the nearest 0.1 kg (without shoes and in light clothing with pockets emptied). The method of measuring body composition via bioelectrical impedance analysis has been previously validated and used in similar clinical studies [30].

### 2.7. Statistical Analysis

Results are presented as the median (25th percentile, 75th percentile) for continuous variables and as number (%) for categorical data. The normality of each continuous variable was tested using the Shapiro–Wilk test. As normality was not found for almost all variables, statistical tests for continuous variables between two groups were performed using the Mann–Whitney U test. Categorical data were tested with a Fisher’s exact test. Effect size is given as r for continuous variables and as Cramer’s V for categorical data. In this study, statistical power calculations for the cross-sectional analysis were performed post hoc, as prior sample-size calculations for the cross-sectional analysis had not been performed.

We used multivariable logistic regression analysis to test the association of intermediate–high-risk Agile 3+ scores with mJDI12 and skeletal muscle mass, and to test the association of mJDI12 with skeletal muscle mass (75th percentile or higher, stratified by sex). In multivariable logistic regression analysis, a low value of events per variable may reduce the reliability of the results [31]. Hence, for covariates that needed to be adjusted, a logistic regression analysis for the outcome was performed to calculate a propensity score, and the effect of the covariates on the outcomes was adjusted by imputing one propensity score as a covariate [32]. The following factors that may affect dietary habits and the Agile 3+ score were used to calculate the propensity score for intermediate–high-risk Agile 3+ scores: sex, age, BMI, diabetes mellitus, hypertension, dyslipidemia, alcohol intake, and medication status (ursodeoxycholic acid, calcium channel blocker, angiotensin receptor blocker, diuretic agent, hydroxymethylglutaryl-coenzyme A reductase inhibitor, tocopherol acetate, dipeptidyl peptidase-4 inhibitor, sodium-glucose cotransporter 2 inhibitor, biguanide, and hypouricemic agent). To calculate propensity scores at or above the 75th percentile for skeletal muscle mass, we used the factors of sex, age, BMI, diabetes mellitus, hypertension, dyslipidemia, and alcohol intake, which are thought to influence dietary habits and skeletal muscle mass. The association between mJDI12 and nutrient intake was examined by multiple regression analysis, stratified by sex. Age and BMI were adjusted as covariates. Because of the exploratory nature of this study, we did not consider a multiplicity of tests. All statistical tests were two-tailed, and *p* < 0.05 was considered statistically significant. Statistical analyses were performed using SPSS ver. 29 (IBM Japan, Tokyo, Japan).

## 3. Results

### 3.1. Patient Characteristics and Laboratory Data Related to Liver Status

The basic characteristics of the patients and laboratory data related to their liver status are presented in Table 1. The number of male and female participants in the study population was about the same. There were 46 patients (34%) with intermediate–high-risk Agile 3+ scores.

### 3.2. Patient Characteristics Grouped by the Agile 3+ Score Risk

Basic patient characteristics were compared between the low-risk and the intermediate–high-risk Agile 3+ score groups (Table 2). The intermediate–high-risk Agile 3+ score group had a significantly higher median age of 15 years and significantly more patients with diabetes and hypertension than the low-risk group. In addition, there was a higher percentage of users of diabetes and hypertension medications in the intermediate–high-risk Agile 3+ score group than in the low-risk group.

### 3.3. Comparison of mJDI12 and its Component Intake in Patients, Grouped by the Agile 3+ Score Risk

There was no significant difference in mJDI12 between the low-risk and the intermediate–high-risk Agile 3+ score groups (Table 3). The intake per 1000 kcal of foods or food groups comprising the mJDI12 was significantly higher for pickles and significantly lower for seaweed in the intermediate–high-risk Agile 3+ score group than in the low-risk Agile 3+ score group.

### 3.4. Relationship between mJDI12 and Nutrient Intake

The mJDI12 showed significant positive associations with protein, potassium, magnesium, iron, folate, vitamin C, β-carotene, α-tocopherol, and dietary fiber intake in patients of both male and female sex. The mJDI12 also showed significant positive associations with calcium, cholesterol, and salt in female, but not male, group (Supplementary Material Table S1).

### 3.5. Association between Intermediate–High-Risk Agile 3+ Scores, and mJDI12 and mJDI12 Components

The association between intermediate–high-risk Agile 3+ scores and mJDI12 and its components was tested by multivariable logistic regression analysis (Table 4). When mJDI12 was used as a continuous variable, mJDI12 had significantly lower odds of intermediate–high-risk Agile 3+ scores (*p* = 0.037). Having an mJDI12 at the 75th percentile or higher (stratified by sex) was associated with significantly lower odds of an intermediate–high-risk Agile 3+ score (*p* = 0.038).

The association between the intake of foods or food groups comprising the mJDI12 and the intermediate–high-risk Agile 3+ score was also examined; the intake of soybeans and soybean foods, fish and shellfish, and seaweeds at or above the 75th percentile, was associated with significantly lower odds of intermediate–high-risk Agile 3+ scores. Seaweeds showed similar results with continuous variables (Supplementary Material Table S2).

### 3.6. Relationship between Intermediate–High-Risk Agile 3+ scores and Skeletal Muscle Mass

There was no significant association with intermediate–high-risk Agile 3+ scores when skeletal muscle mass was entered as a continuous variable. When skeletal muscle mass was stratified by sex, skeletal muscle mass at the 75th percentile or higher had significantly lower odds of intermediate–high-risk Agile 3+ scores (*p* = 0.022) (Table 5).

### 3.7. Relationship between Skeletal Muscle Mass, and mJDI12 and its Components

As skeletal muscle mass at the 75th percentile or higher was a factor significantly associated with intermediate–high-risk Agile 3+ scores, the association of mJDI12 for skeletal muscle mass at the 75th percentile or higher was tested by logistic regression analysis (Table 6). The mJDI12 was not a factor significantly associated with skeletal muscle mass for continuous variables (*p* = 0.18), or for the 75th percentile or higher (*p* = 0.12).

For the mJDI12 components, the intake of soybeans and soybean foods was a significant factor associated with skeletal muscle mass at the 75th percentile or higher (*p* = 0.049) (Supplementary Material Table S3).

## 4. Discussion

In this study, we reveal that a daily diet following the Japanese diet pattern was significantly associated with a lower risk of advanced fibrosis in patients with NAFLD. Among the Japanese diet components, a higher intake of soybeans and soybean foods, fish and shellfish, and seaweeds was associated with a lower risk of advanced fibrosis. To the best of our knowledge, this is the first report to reveal that the Japanese diet pattern is associated with the severity of liver fibrosis in patients with NAFLD.

The Mediterranean diet pattern may be effective as a diet for patients with NASH [14], and the Japanese diet pattern might also be associated with a lower risk of liver fibrosis. The Japanese diet pattern is a food pattern that consists mainly of fish and shellfish, and is rich in high-fiber foods, such as seaweeds and mushrooms, and fermented foods, such as pickles. Intervention with a Japanese diet pattern improved lipid metabolism in young adults [16]. In addition, a Japanese diet pattern score has been associated with lower obesity rates and ischemic heart disease incidence in a global comparative study [33]. Interestingly, we found that mJDI12 was also significantly associated with the intake of nutrients with antioxidant properties, such as vitamin C, β-carotene, and α-tocopherol. Oxidative stress is associated with the pathogenesis of NASH [34], and intervention with antioxidant nutrients, such as vitamin E, may have a hepatoprotective effect [35]. It may be possible that results were consistent with our results in Supplementary Material Table S1 and Table 4. Although the effect of dietary fiber intake on progression of NAFLD has not been fully studied in humans [36], the Japanese diet pattern may be associated with a lower risk of the progression of NAFLD, because of the reported cholesterol-lowering effects and improvement of insulin resistance from high dietary fiber intake [37]. In addition, the association between the Japanese diet pattern and the risk of liver fibrosis may be influenced by the intestinal microbiota, because dietary fiber affects the intestinal microbiota and the intestinal environment [37], and because there may be an association between the intestinal microbiota and the intestinal environment, and the pathogenesis of NASH [38].

In this study, there was a significant association between a low risk of advanced liver fibrosis and high skeletal muscle mass after adjustment for confounders such as age, sex, and BMI. Patients with cirrhosis are prone to skeletal muscle loss caused by various pathological effects [39]. An association between liver fibrosis or steatohepatitis and sarcopenia has been reported in patients with NAFLD [40], and the results obtained in the present study on the association between the risk of liver fibrosis and skeletal muscle mass imply that skeletal muscle mass is low as a result of advanced liver fibrosis. In addition, skeletal muscle plays an important role in glucose metabolism, and it has been reported that impaired glucose tolerance is higher in patients with low skeletal muscle mass [17]. Furthermore, because it has been reported that low skeletal muscle mass is associated with the development and pathological progression of NAFLD in a longitudinal study [41], the relationship between skeletal muscle mass and liver fibrosis may be reciprocal.

In our survey of the association between skeletal muscle mass and mJDI12, we found that mJDI12 was not significantly associated with skeletal muscle mass. However, soybeans and soybean food intake was a factor significantly associated with having a skeletal muscle mass at the 75th percentile or higher. As soy protein has been reported to increase skeletal muscle mass gain more than casein—a milk protein—in subjects with low physical activity [42], it would be interesting to further analyze data from patients that eat a diet high in soybeans and soybean foods in conjunction with an analysis of physical activity levels. In dietary interventions for patients with NAFLD, it has been reported that reduced protein intake, when reducing energy intake, is associated with decreased skeletal muscle mass [43]. Maintaining an intake of soybeans and soybean foods as a source of protein may be important for the maintenance of skeletal muscle mass.

We showed that soybeans and soybean food intake was associated with a lower risk of liver fibrosis and greater skeletal muscle mass. Although genistein, an isoflavone abundant in soybeans, did not decrease aspartate aminotransferase and alanine aminotransferase in patients with NAFLD after 8 weeks of intake, it did improve body fat loss, decrease the waist–hip ratio, and reduce blood triglyceride levels [44], suggesting that genistein inhibits the development of NAFLD. In addition, the soy protein, β-conglycinin, may reduce the risk of NASH, as it inhibited NASH progression in a mouse model of NASH [45]. Thus, soy-specific components may have a combined hepatoprotective effect on lipid metabolism in the liver and on skeletal muscle mass.

There are limitations to this study that should be considered. The first limitation is that our study was cross-sectional. Dietary patterns may change as a result of disease states; therefore, our results need to be validated in prospective and intervention studies. Because our study is a prospective cohort study, we plan to examine changes in the Japanese diet pattern and the risk of advanced liver fibrosis in our patients. The second limitation is that the risk of advanced liver fibrosis was evaluated using the Agile 3+ score. A more accurate assessment of advanced liver fibrosis requires evaluation by liver biopsy. However, liver biopsy evaluations vary by pathologist and have very poor inter-reader variability and modest intra-reader variability [46]. The third limitation is that our patients were recruited from a single institution. Patient characteristics and treatment conditions may differ among hospitals; therefore, a multicenter study is needed to generalize the results of this study. The fourth limitation is the method used to calculate the mJDI12. The mJDI12 was calculated from the median intake of 12 foods or food groups in our study population, so the median may vary depending on the characteristics of the subjects. It would be desirable to calculate the median intake in subjects who are non-obese and not at risk of fatty liver, and to calculate the mJDI12 in patients with NAFLD based on that median value. However, associations between mJDI12 and the intake of several foods or food groups were able to be observed when mJDI12 was calculated using data from our study population of patients with NAFLD.

## 5. Conclusions

The Japanese diet pattern and skeletal muscle mass may be associated with the severity of liver fibrosis in patients with NAFLD, and soybeans and soybean food intake may affect skeletal muscle mass.

## Figures and Tables

**Table 1:** Study subject characteristics and laboratory data related to liver status.

Number of the Patients	136
Female	67 (49)
Age (years)	60 (50, 70)
BMI (kg/m^2^)	26.8 (23.9, 29.8)
Drinking habits	62 (46)
**Body composition**	
Skeletal muscle mass (kg)	24.7 (21.1, 29.9)
Percent body fat (%)	33.9 (27.8, 39.0)
**FibroScan data**	
Agile 3+ score	0.29 (0.09, 0.61)
Agile 3+ score risk (low/intermediate/high)	90 (66)/19 (14)/27 (20)
Liver stiffness measurement (kPa)	6.35 (4.33, 9.70)
Controlled attenuation parameter (dB/m)	281 (248, 317)
**Laboratory data**	
AST (U/L)	32 (24, 44)
ALT (U/L)	37 (23, 64)
Platelets (10^4^/μL)	22.4 (18.4, 26.9)
**Comorbidity**	
Diabetes mellitus	54 (40)
Hypertension	55 (40)
Dyslipidemia	86 (63)
**Medication status**	
Ursodeoxycholic acid	29 (21)
Calcium channel blocker	30 (22)
Angiotensin receptor blocker	32 (24)
Diuretic agent	8 (6)
HMG-CoA reductase inhibitor	30 (22)
Tocopherol acetate	27 (20)
Dipeptidyl peptidase-4 inhibitor	35 (26)
Sodium-glucose cotransporter 2 inhibitor	29 (21)
Biguanide	12 (9)
Hypouricemic agent	25 (18)

Data are shown as the median (25th, 75th percentile) or number (%). BMI, body mass index; AST, aspartate aminotransferase; ALT, alanine aminotransferase; HMG-CoA, hydroxymethylglutaryl-coenzyme A.

**Table 2:** Subject characteristics and laboratory data related to liver status, grouped by the Agile 3+ score risk.

	Low-Risk Agile 3+ Scores	Intermediate–High-Risk Agile 3+ Scores	*p* Value	Effect Size
Number of the patients	90	46	-	-
Female	41 (46)	26 (57)	0.28	0.10
Age (years)	57 (48, 64)	72 (63, 75)	<0.001	0.43
BMI (kg/m^2^)	26.3 (23.3, 28.7)	28.0 (25.0, 31.2)	0.02	0.20
Drinking habits	46 (51)	16 (35)	0.10	0.16
**Body composition**				
Skeletal muscle mass (kg)	24.9 (21.3, 30.2)	23.5 (20.7, 28.8)	0.30	0.09
Percent body fat (%)	32.4 (26.9, 39.1)	35.9 (31.1, 38.9)	0.15	0.12
**FibroScan data**				
Agile 3+ score	0.15 (0.06, 0.28)	0.83 (0.59, 0.96)	<0.001	0.82
Liver stiffness measurement (kPa)	5.10 (4.00, 7.10)	12.3 (7.2, 19.3)	<0.001	0.64
Controlled attenuation parameter (dB/m)	285 (247, 318)	271 (248, 314)	0.36	0.08
**Laboratory data**				
AST (U/L)	29 (23, 36)	32 (22, 68)	0.005	0.24
ALT (U/L)	39 (24, 62)	32 (22, 68)	0.47	0.06
Platelets (10^4^/μL)	25.0 (20.0, 28.8)	18.0 (14.1, 22.9)	<0.001	0.48
**Comorbidity**				
Diabetes mellitus	24 (27)	30 (65)	<0.001	0.37
Hypertension	24 (27)	31 (67)	<0.001	0.39
Dyslipidemia	60 (67)	26 (57)	0.26	0.10
**Medication status**				
Ursodeoxycholic acid	16 (18)	13 (28)	0.19	0.12
Calcium channel blocker	12 (13)	18 (39)	0.001	0.29
Angiotensin receptor blocker	15 (17)	17 (37)	0.011	0.23
Diuretic agent	1 (1)	7 (15)	0.002	0.28
HMG-CoA reductase inhibitor	20 (22)	10 (22)	1.000	0.01
Tocopherol acetate	12 (13)	15 (33)	0.012	0.23
Dipeptidyl peptidase-4 inhibitor	13 (14)	22 (48)	<0.001	0.36
Sodium-glucose cotransporter 2 inhibitor	13 (14)	16 (35)	0.008	0.24
Biguanide	5 (6)	7 (15)	0.11	0.16
Hypouricemic agent	16 (18)	9 (20)	0.82	0.02

Data are shown as the median (25th, 75th percentile) or number (%). Statistical tests of continuous variables between the two groups were performed by a Mann–Whitney U test. Categorical data were tested by a Fisher’s exact test. In the Mann–Whitney U test, the alpha error was less than 0.05 and power was greater than 0.8 when the effect size was greater than 0.53. In Fisher’s exact test, the alpha error was less than 0.05 and power was greater than 0.8 when the effect size was greater than 0.25.

**Table 3:** Comparison of mJDI12 and its components, grouped by the Agile 3+ score risk.

	Low-Risk Agile 3+ Scores	Intermediate–High-Risk Agile 3+ Scores	*p* Value	Effect Size
mJDI12	6.0 (4.0, 8.0)	6.0 (4.0, 7.3)	0.80	0.02
Soybeans and soybean foods	25.4 (10.7, 43.5)	22.3 (9.9, 38.5)	0.33	0.08
Green and yellow vegetables	45.0 (29.1, 75.3)	61.1 (20.1, 80.8)	0.45	0.07
Fruit	46.5 (19.5, 86.3)	64.0 (31.7, 103.7)	0.17	0.12
Fish and shellfish	36.9 (24.2, 58.8)	39.5 (23.0, 62.5)	0.85	0.02
Pickles	2.9 (0.7, 9.1)	5.3 (2.1, 11.2)	0.021	0.2
Mushrooms	6.0 (2.2, 9.5)	4.8 (2.3, 11.0)	0.79	0.02
Seaweeds	4.1 (1.8, 9.8)	2.4 (1.5, 6.6)	0.013	0.21
Green tea	91.2 (24.1, 290.0)	180.2 (76.2, 288.2)	0.39	0.07
Rice	130.8 (93.3, 199.2)	137.3 (72.1, 167.1)	0.58	0.05
Miso soup	55.8 (34.8, 81.1)	42.6 (30.2, 77.1)	0.12	0.14
Beef and pork	19.4 (13.4, 29.3)	21.4 (10.6, 32.8)	0.61	0.04
Coffee	87.9 (20.2, 213.2)	89.8 (48.1, 194.6)	0.66	0.04

Data are shown as the median (25th, 75th percentile) intake per 1000 kcal. Statistical tests of continuous variables between the two groups were performed by a Mann–Whitney U test. Categorical data were tested by a Fisher’s exact test. In the Mann–Whitney U test, the alpha error was less than 0.05 and power was greater than 0.8 when the effect size was greater than 0.53. mJDI12; 12-component modified Japanese diet index.

**Table 4:** Logistic regression analysis of mJDI12 for intermediate–high-risk Agile 3+ scores.

		OR	95% CI	*p* Value
model 1	mJDI12	0.77	0.61, 0.99	0.04
model 2	<25 percentile (reference)	-	-	
	≥25 to <50 percentile	0.31	0.06, 1.56	0.16
	≥50 to <75 percentile	0.24	0.04, 1.35	0.11
	≥75 percentile	0.17	0.03, 0.90	0.038

In logistic regression analysis with intermediate–high-risk Agile 3+ score as the outcome, sex, age, BMI, diabetes mellitus, hypertension, dyslipidemia, alcohol intake, and medication status (ursodeoxycholic acid, calcium channel blocker, angiotensin receptor blocker, diuretic agent, HMG-CoA reductase inhibitor, tocopherol acetate, dipeptidyl peptidase-4 inhibitor, sodium-glucose cotransporter 2 inhibitor, biguanide, and hypouricemic agent) were included as covariates, and the propensity score was calculated. We tested the association between mJDI12 and intermediate–high-risk Agile 3+ scores in a model with the calculated propensity score as one variable in the covariate. In model 1, mJDI12 was imputed as a crude value. In model 2, mJDI12 was imputed as a quartile. OR, odds ratio; CI, confidence interval.

**Table 5:** Logistic regression analysis of skeletal muscle mass for Agile 3+ score intermediate–high risk.

		OR	95% CI	*p* Value
model 1	Skeletal muscle mass	1.01	0.94, 1.09	0.78
model 2	<25 percentile (reference)	-	-	
	≥25 to <50 percentile	0.64	0.15, 2.67	0.54
	≥50 to <75 percentile	0.55	0.12, 2.60	0.45
	≥75 percentile	0.23	0.07, 0.77	0.017

In logistic regression analysis with Agile3+ score intermediate–high risk as outcome, sex, age, BMI, diabetes mellitus, hypertension, dyslipidemia, alcohol intake, the medication status (ursodeoxycholic acid, calcium channel blocker, angiotensin receptor blocker, diuretic agent, HMG-CoA reductase inhibitor, tocopherol acetate, dipeptidyl peptidase-4 inhibitor, sodium–glucose cotransporter 2 inhibitor, biguanide, and hypouricemic agent) were included as covariates, and the propensity score was calculated. We tested the association between skeletal muscle mass and Agile 3+ score intermediate–high risk in a model with the calculated propensity score as one variable in the covariate. In model 1, skeletal muscle mass was imputed as a crude value. In model 2, skeletal muscle mass was imputed as a sex-specific quartile. OR; odds ratio, CI; confidence interval; BMI, body mass index; HMG-CoA, hydroxymethylglutaryl-coenzyme A.

**Table 6:** Logistic regression analysis of mJDI12 for skeletal muscle mass at the 75th percentile or higher.

		OR	95% CI	*p* Value
model 1	mJDI12	1.15	0.94, 1.40	0.18
model 2	<25 percentile (reference)	-	-	
	≥25 to <50 percentile	3.86	0.69, 21.64	0.12
	≥50 to <75 percentile	3.13	0.53, 18.33	0.21
	≥75 percentile	3.99	0.69, 22.99	0.12

In logistic regression analysis for skeletal muscle mass at the 75th percentile or higher, sex, age, BMI, diabetes mellitus, hypertension, dyslipidemia, and alcohol intake were included as covariates, and the propensity score was calculated. We tested the association between mJDI12 and skeletal muscle mass at the 75th percentile or higher, in a model with the calculated propensity score as one variable in the covariate. In model 1, mJDI12 was imputed as a crude value. In model 2, mJDI12 was imputed as a sex-specific quartile.

## Data Availability

The data presented in this study are available on request from the corresponding author. The data are not publicly available due to ethical aspects.

## Supplementary Materials

The following supporting information can be downloaded at: https://www.mdpi.com/article/10.3390/nu15051175/s1. Table S1: Association between mJDI12 and nutrient intake in study subjects. Table S2: Logistic regression analysis of mJDI12 components for Agile 3+ score intermediate–high risk. Table S3: Logistic regression analysis of mJDI12 components for skeletal muscle mass 75th percentile or higher.
