# Supplementary material for: Severity of Liver Fibrosis Is Associated with the Japanese Diet Pattern and Skeletal Muscle Mass in Patients with Nonalcoholic Fatty Liver Disease
Source: Nutrients. 2023 Feb 26;15(5):1175. doi: 10.3390/nu15051175 (PMC10005291; doi:10.3390/nu15051175)
Supplement: Supplementary file 1 [file nutrients-15-01175-s001.zip › nutrients-2233295-supplementary.pdf]

Table S1 Association between mJDI12 and nutrients intake in study subjects

	Male				Female			
	β	B	95% CI	p value	*β*	B	95% CI	p value
Protein	0.27	1.09	0.45, 1.74	0.001	0.32	1.33	0.34, 2.32	0.009
Fat	-0.05	-0.14	-0.93, 0.65	0.73	-0.05	-0.15	-0.93, 0.64	0.71
Carbohydrate	-0.10	-0.96	-3.41, 1.49	0.44	-0.05	-0.52	-3.00, 1.96	0.68
Potassium	0.38	64.23	23.70, 104.76	0.002	0.51	86.74	51.58, 121.90	<0.001
Calcium	0.24	10.49	-0.17, 21.14	0.05	0.33	15.18	4.72, 25.65	0.005
Magnesium	0.46	5.76	2.87, 8.65	<0.001	0.54	7.94	4.89, 10.98	<0.001
Iron	0.52	0.27	0.15, 0.39	<0.001	0.64	0.35	0.25, 0.46	<0.001
Folic acid	0.47	14.83	7.48, 22.18	<0.001	0.63	19.34	13.40, 25.25	<0.001
Vitamin C	0.47	5.75	2.94, 8.56	<0.001	0.56	7.3	4.70, 9.90	<0.001
β-carotene	0.52	263.2	147.30, 379.09	<0.001	0.52	244.2	144.22, 344.18	<0.001
α-tocopherol	0.33	0.16	0.04, 0.28	0.009	0.35	0.18	0.06, 0.29	0.004
SFA	-0.12	-0.13	-0.42, 0.15	0.36	-0.17	-0.15	-0.37, 0.07	0.18
MUFA	-0.1	-0.12	-0.43, 0.18	0.43	-0.13	-0.17	-0.49, 0.16	0.31
PUFA	0.08	0.06	-0.13, 0.25	0.54	0.1	0.1	-0.15, 0.34	0.43
n-3 PUFA	0.24	0.05	-0.002, 0.11	0.06	0.23	0.06	-0.003, 0.12	0.06
n-6 PUFA	0.01	0.01	-0.15, 0.16	0.95	0.05	0.04	-0.16, 0.23	0.70
Cholesterol	0.25	10.48	-0.26, 21.21	0.06	0.28	12.96	1.84, 24.09	0.02
Dietary fiber	0.48	0.39	0.21, 0.58	<0.001	0.63	0.54	0.38, 0.71	<0.001
Salt equivalent	0.17	0.11	-0.06, 0.28	0.20	0.32	0.21	0.06, 0.36	0.008

The association of each data was analyzed by multiple regression analysis. Age, BMI were adjusted. B; partial regression coefficient, β; standardized partial regression coefficient, CI; confidence interval, mJDI12; 12-component modified Japanese diet index, MUFA; monounsaturated fatty acids, PUFA; polyunsaturated fatty acids, RA; rheumatoid arthritis, SFA; saturated fatty acids

Table S2 Logistic regression analysis of mJDI12 components for Agile3+ score intermediate-high risk

		OR	95%CI	p value
model 1	Soybeans and soybean foods	0.98	0.96, 1.01	0.11
model 2	<25 percentile (reference)	-	-	
	≥25 to <50 percentile	0.41	0.11, 1.57	0.19
	≥50 to <75 percentile	0.40	0.10, 1.65	0.20
	≥75 percentile	0.15	0.03, 0.71	0.017
model 1	Green and yellow vegetables	1.00	0.99, 1.02	0.96
model 2	<25 percentile (reference)	-	-	
	≥25 to <50 percentile	0.12	0.02, 0.62	0.012
	≥50 to <75 percentile	0.50	0.13, 1.91	0.31
	≥75 percentile	0.40	0.10, 1.64	0.20
model 1	Fruit	1.00	0.99, 1.01	0.52
model 2	<25 percentile (reference)	-	-	
	≥25 to <50 percentile	0.45	0.11, 1.84	0.27
	≥50 to <75 percentile	0.54	0.14, 2.11	0.38
	≥75 percentile	0.47	0.12, 1.91	0.29
model 1	Fish and shellfish	0.99	0.97, 1.01	0.14
model 2	<25 percentile (reference)	-	-	
	≥25 to <50 percentile	0.57	0.14, 2.24	0.42
	≥50 to <75 percentile	0.14	0.03, 0.70	0.018
	≥75 percentile	0.20	0.04, 0.95	0.043
model 1	Pickles	0.98	0.92, 1.05	0.56
model 2	<25 percentile (reference)	-	-	
	≥25 to <50 percentile	2.06	0.48, 8.91	0.33
	≥50 to <75 percentile	1.97	0.46, 8.41	0.36
	≥75 percentile	2.94	0.69, 12.53	0.15
model 1	Mushrooms	0.95	0.85, 1.06	0.37
model 2	<25 percentile (reference)	-	-	
	≥25 to <50 percentile	0.68	0.18, 2.61	0.58
	≥50 to <75 percentile	0.59	0.14, 2.43	0.47
	≥75 percentile	0.30	0.07, 1.29	0.11
model 1	Seaweeds	0.85	0.76, 0.95	0.005
model 2	<25 percentile (reference)	-	-	
	≥25 to <50 percentile	1.11	0.28, 4.45	0.88

	≥50 to <75 percentile	0.07	0.01, 0.40	0.003
	≥75 percentile	0.08	0.01, 0.44	0.004
model 1	Green tea	1.00	1.00, 1.00	0.91
model 2	<25 percentile (reference)	-	-	
	≥25 to <50 percentile	1.20	0.31, 4.64	0.79
	≥50 to <75 percentile	1.53	0.39, 6.01	0.55
	≥75 percentile	0.82	0.21, 3.22	0.78
model 1	Rice	1.00	1.00, 1.01	0.33
model 2	<25 percentile (reference)	-	-	
	≥25 to <50 percentile	0.15	0.03, 0.77	0.022
	≥50 to <75 percentile	1.52	0.40, 5.84	0.54
	≥75 percentile	1.19	0.28, 5.08	0.82
model 1	Miso soup	0.99	0.98, 1.00	0.18
model 2	<25 percentile (reference)	-	-	
	≥25 to <50 percentile	0.31	0.08, 1.22	0.09
	≥50 to <75 percentile	0.28	0.07, 1.11	0.07
	≥75 percentile	0.27	0.07, 1.10	0.07
model 1	Beef and pork	1.01	0.97, 1.05	0.61
model 2	<25 percentile (reference)	-	-	
	≥25 to <50 percentile	0.36	0.09, 1.49	0.16
	≥50 to <75 percentile	0.63	0.16, 2.45	0.51
	≥75 percentile	1.18	0.31, 4.55	0.81
model 1	Coffee	1.00	1.00, 1.01	0.53
model 2	<25 percentile (reference)	-	-	
	≥25 to <50 percentile	0.34	0.07, 1.64	0.18
	≥50 to <75 percentile	0.66	0.16, 2.72	0.56
	≥75 percentile	0.63	0.15, 2.59	0.52

In logistic regression analysis with intermediate–high-risk Agile 3+ score as the outcome, sex, age, BMI, diabetes mellitus, hypertension, dyslipidemia, alcohol intake, and medication status (ursodeoxycholic acid, calcium channel blocker, angiotensin receptor blocker, diuretic agent, HMG-CoA reductase inhibitor, tocopherol acetate, dipeptidyl peptidase-4 inhibitor, sodium-glucose cotransporter 2 inhibitor, biguanide, and hypouricemic agent) were included as covariates and the propensity score was calculated. We tested the association between mJDI12 components and intermediate–high-risk Agile 3+ scores in independent models with the calculated propensity score as one variable in the covariate. In model 1, mJDI12 components were imputed as a crude value. In model 2, mJDI12 components were imputed as a quartile. OR, odds ratio; CI, confidence interval.

Table S3 Logistic regression analysis of mJDI12 components for skeletal muscle mass 75th percentile or higher

		OR	95%CI	p value
model 1	Soybeans and soybean foods	1.02	1.00, 1.04	0.049
model 2	<25 percentile (reference)	-	-	
	≥25 to <50 percentile	1.86	0.56, 6.13	0.31
	≥50 to <75 percentile	1.93	0.59, 6.27	0.28
	≥75 percentile	3.07	0.95, 9.91	0.06
model 1	Green and yellow vegetables	1.00	0.98, 1.01	0.42
model 2	<25 percentile (reference)	-	-	
	≥25 to <50 percentile	0.60	0.19, 1.88	0.38
	≥50 to <75 percentile	1.15	0.38, 3.49	0.81
	≥75 percentile	0.76	0.24, 2.40	0.64
model 1	Fruit	1.00	1.00, 1.01	0.41
model 2	<25 percentile (reference)	-	-	
	≥25 to <50 percentile	3.72	1.09, 12.68	0.036
	≥50 to <75 percentile	2.31	0.71, 7.51	0.17
	≥75 percentile	2.93	0.86, 9.98	0.09
model 1	Fish and shellfish	1.00	0.98, 1.01	0.61
model 2	<25 percentile (reference)	-	-	
	≥25 to <50 percentile	1.01	0.33, 3.13	0.99
	≥50 to <75 percentile	1.50	0.50, 4.56	0.47
	≥75 percentile	1.13	0.36, 3.58	0.84
model 1	Pickles	0.99	0.94, 1.05	0.84
model 2	<25 percentile (reference)	-	-	
	≥25 to <50 percentile	0.95	0.31, 2.87	0.92
	≥50 to <75 percentile	1.19	0.40, 3.57	0.76
	≥75 percentile	0.58	0.18, 1.87	0.36
model 1	Mushrooms	1.01	0.94, 1.08	0.77
model 2	<25 percentile (reference)	-	-	
	≥25 to <50 percentile	0.46	0.14, 1.58	0.22
	≥50 to <75 percentile	1.49	0.48, 4.68	0.49
	≥75 percentile	1.52	0.50, 4.66	0.46
model 1	Seaweeds	1.02	0.96, 1.07	0.61
model 2	<25 percentile (reference)	-	-	
	≥25 to <50 percentile	0.96	0.29, 3.23	0.95

	≥50 to <75 percentile	2.97	0.92, 9.52	0.07
	≥75 percentile	1.40	0.43, 4.58	0.58
model 1	Green tea	1.00	1.00, 1.00	0.90
model 2	<25 percentile (reference)	-	-	
	≥25 to <50 percentile	0.85	0.28, 2.64	0.78
	≥50 to <75 percentile	1.15	0.38, 3.48	0.81
	≥75 percentile	0.99	0.32, 3.05	0.99
model 1	Rice	1.00	1.00, 1.01	0.93
model 2	<25 percentile (reference)	-	-	
	≥25 to <50 percentile	0.99	0.33, 2.99	0.99
	≥50 to <75 percentile	0.36	0.11, 1.25	0.11
	≥75 percentile	1.09	0.36, 3.28	0.88
model 1	Miso soup	1.00	0.99, 1.00	0.26
model 2	<25 percentile (reference)	-	-	
	≥25 to <50 percentile	1.26	0.41, 3.85	0.69
	≥50 to <75 percentile	0.61	0.20, 1.91	0.40
	≥75 percentile	0.93	0.30, 2.87	0.90
model 1	Beef and pork	0.99	0.95, 1.02	0.35
model 2	<25 percentile (reference)	-	-	
	≥25 to <50 percentile	0.60	0.20, 1.83	0.37
	≥50 to <75 percentile	0.69	0.23, 2.08	0.50
	≥75 percentile	0.51	0.16, 1.59	0.25
model 1	Coffee	1.00	1.00, 1.00	0.69
model 2	<25 percentile (reference)	-	-	
	≥25 to <50 percentile	1.96	0.61, 6.25	0.26
	≥50 to <75 percentile	0.75	0.23, 2.43	0.63
	≥75 percentile	1.74	0.54, 5.61	0.35

In logistic regression analysis for sex specific skeletal muscle mass 75th percentile or higher, sex, age, BMI, diabetes mellitus, hypertension, dyslipidemia, alcohol intake, were included as covariates and propensity score was calculated. We tested the association between mJDI12 components and skeletal muscle mass 75th percentile of more in a model with the calculated propensity score as one variable in the covariate. In model 1, each mJDI12 component was imputed as crude value. In model 2, each mJDI12 component was imputed as a sex specific quartile. OR; odds ratio, CI; confidence interval; mJDI12; 12-component modified Japanese diet index, BMI; body mass index
